# An Insight Into Various Manifestations of Leptospirosis: A Unique Case Series From a State in Eastern India

**DOI:** 10.7759/cureus.56802

**Published:** 2024-03-24

**Authors:** Divakar Kumar, Manohar Lal Prasad, Mukesh Kumar, Shiv S Munda, Vidyapati

**Affiliations:** 1 Internal Medicine, Rajendra Institute of Medical Sciences, Ranchi, IND; 2 Community Medicine, Phulo Jhano Medical College, Dumka, IND

**Keywords:** antibiotics, clinical manifestation, microscopic agglutination test, case-series, atypical presentation, febrile illness, leptospirosis

## Abstract

Background: Leptospirosis presents with highly variable clinical manifestations affecting different organ systems in different individuals. The presentation ranges from an asymptomatic or mild disease to a severe disease associated with multiorgan failure and higher mortality. Leptospirosis is highly underreported due to a lack of diagnostic modalities and less suspicion among clinicians.

Methodology: We present this single-center retrospective case series of 12 cases, which include various common and uncommon scenarios by which the disease can present and can be missed due to lack of suspicion. The study contains individual patient characteristics including demographic, laboratory, clinical, and treatment data. The association between these variables and mortality was analyzed using p-values and results were described. A p-value of<0.05 was considered statistically significant.

Results: A total of 12 cases were included in the study. The male-to-female ratio was 3:1. The mean age was higher (37.75±9.81 years) in cases who died than those who recovered (34.25±14.09). Factors like history of alcoholism, presence of chronic liver disease (CLD), jaundice, acute renal failure, requirement of dialysis, and requirement of intensive care were significantly associated with increased risk of death (odds ratio >1, p-value <0.05). The most common symptom of presentation was fever in 11 (91.66%) cases. Jaundice and renal failure were significantly associated with death (odds ratio 1.2, p-value 0.04). The requirement of intensive care treatment (odds ratio 2.1, p-value 0.05) and dialysis (odds ratio 39.66, p-value 0.03) were also significantly associated with death. The percentage of death was lower in the group of patients who received combination antibiotic therapy.

Conclusion: Leptospirosis has varied presentations in different individuals and the diagnosis can be missed due to lack of specific signs and symptoms. Severe diseases involving multiple organs and preexisting comorbidities are associated with higher mortality rates. Timely diagnosis and treatment are necessary to reduce mortality and increase survival.

## Introduction

Leptospirosis is a bacterial disease that occurs all over the world in varying frequency caused by the pathogenic strains of bacteria of the genus Leptospira. It is a zoonotic disease affecting both humans and animals. The clinical spectrum of leptospirosis is very wide with mild anicteric presentation at one end to severe leptospirosis with severe jaundice and multiple organ involvement on the other end [1]. Fever, nausea, vomiting, myalgia, and headache are some of the non-specific vague symptoms in mild leptospirosis, while Weil’s syndrome, a severe form of leptospirosis involves multiple organs like the liver, kidney, lungs, and vascular system [1]. Transmission of the leptospiral bacteria usually occurs through the urine of infected animals which can get into soil and water and enter the human body through broken skin like a scratch or cut or through the mucosa of the oral cavity, nose, and eyes [2]. This is the most common mode of transmission. Other less common and rare modes of transmission are direct contact with infected animals, placental transmission, bite by an infected animal, through the lung by inhalation of aerosol containing pathogenic Leptospira, consumption of contaminated water or food, and sodden and waterlogged skin [2].

Pathogenesis of leptospiral infection in humans occurs in two phases, leptospiremic or septicemic phase and immune phase. Once pathogenic Leptospira is inside the body by penetrating the tissue barriers and evading the innate immune system, it starts multiplying and proliferating and gains access to the circulatory system. This is the start of the septicemic and leptospiremic phase in which symptoms are mild and nonspecific like fever, body ache, headache, nausea, and vomiting. This stage lasts for approximately eight days from the onset of the fever. Once in the circulatory system, it disseminates very rapidly by hematogenous route mainly capillaries to different organ systems [2]. Leptospiral bacteria cause capillary vasculitis and capillary leakage which is the main reason for the damage of different organ systems [3]. The immune phase starts with the involvement of different organ systems and, there is the appearance of antibodies in the blood and bacteria start to disappear from blood [1,2]. In the immune phase, numerous cytokines predominantly IL-6, TNF-a, and IL-10 appear in the blood to fight infection. Sometimes there can be an overwhelming number of cytokines causing a sepsis and cytokine storm-like presentation which can be fatal [1]. Among different organ systems, the liver is most commonly involved. In the liver, it causes hepatocellular dysfunction, centrilobular necrosis, and Kupffer cell proliferation leading to raised bilirubin and liver enzymes. In the kidney, it can cause interstitial nephritis and hypovolemic prerenal failure. In the lungs, it causes alveolar hemorrhage due to the involvement of vessels in the alveoli and interstitium, which is one of the most common causes of death in leptospirosis. It can cause pancreatitis, aseptic meningitis, and interstitial carditis due to the involvement of the pancreas, brain, and heart respectively. Thrombotic thrombocytopenic purpura (TTP), DIC, and HUS may also develop in some patients with leptospirosis [1,2,4].

Diagnosis of leptospirosis is done by confirmatory tests after initial screening. Immunoglobulin M (IgM) enzyme-linked immunosorbent assay is the most commonly used screening test. Confirmatory test for leptospira includes a microscopic agglutination test (MAT), a culture of body fluids or tissue, and DNA polymerase chain reaction (PCR) of CSF, blood, and urine. MAT is the gold standard test used to confirm the diagnosis of leptospirosis, whose titer is 1:400 in a single measurement or more than four times the rise of titer between the first and fourth week of the disease, which is considered diagnostic [2,4]. Treatment of leptospirosis includes antibiotics, oral or intravenous, supportive measures, and treatment of complications. Mild leptospirosis is self-limited and usually requires no treatment or sometimes only oral antibiotics. Intravenous antibiotics are required for severe and hospitalized patients. Leptospiral bacteria are sensitive to different groups of antibiotics including cephalosporins, doxycycline, beta-lactam, fluoroquinolones, and macrolides [2,3]. Early diagnosis, prompt treatment, and absence of comorbidities carry a good prognosis [4]. Despite treatment, some patients have persisting symptoms of fatigue and headache. Diagnosis and treatment of leptospirosis are often delayed due to overlapping signs and symptoms with other common febrile illnesses and a lack of diagnostic modalities. Physicians must be aware of different common, uncommon, and rare ways of presentation of leptospirosis in humans so that early treatment can be done and mortality can be reduced. Hereby we report a case series of leptospirosis which will help physicians in the early diagnosis and treatment of leptospirosis.

## Materials and methods

Case presentation

This is a case series on different clinical presentations of leptospirosis reported from a tertiary care centre in Jharkhand state which has a significant proportion of tribal and rural population. The study was conducted from July 2023 to September 2023 which corresponds to the rainy season in this area. Suspected cases of leptospirosis were analyzed by clinical, laboratory, and radiological features. Routine blood tests like complete blood counts (CBC), renal function tests, liver function tests, serum electrolytes, prothrombin time/International Normalized Ratio (PT/INR), CRP, D-dimer, random blood sugar (RBS), urine analysis, and radiological test X-ray chest, ultrasonography of the abdomen were done for all patients. Serum amylase, lipase, CSF examination, and computed tomography (CT) scan of the brain were also done when required. The cases were screened by enzyme-linked immunosorbent assay (ELISA) titer for leptospira IgM antibodies, and the diagnosis was confirmed by MAT. The cerebrospinal fluid polymerase chain reaction (CSF-PCR) test was used to confirm suspected meningitis cases. Cases with positive IgM antibody test and MAT titer≥1:400 were diagnosed as confirmed cases of leptospirosis. Positive cases were included in the case series which accounted for 12 cases. Ethical committee approval was not required for this study. Our institution does not require ethical committee approval for reporting individual cases or case series-based studies. Informed consent was taken from each patient after explaining the study and their anonymity and confidentiality were maintained throughout the study period and publication process. The management of patients was not affected by the current study.

Statistical analysis

Individual patient characteristics including demographic details, laboratory findings, treatment modalities, and outcomes were recorded in a Microsoft Excel (Microsoft Corporation, Redmond, Washington, USA) sheet. The data were described as continuous or categorical variables. The continuous variables were presented as mean with standard deviation and median with interquartile range (IQR) and categorical variables were presented as numbers and percentages. The outcome was recorded as recovery or death, and the results were summarized in the form of tables and graphs. The chi-square test was used to compare qualitative variables while the Student t-test was used to compare quantitative variables. A p-value of <0.05 was considered statistically significant. The odds ratio was calculated to assess the relationship between exposure to risk factors and outcome variables. The statistical software Statistical Package for Social Sciences (SPSS), version 22.0 (IBM Corp., Armonk, NY) was used for all statistical calculations.

Individual patient characteristics are shown in Table 1.

**Table 1:** Individual patient characteristics. AKI: Acute kidney injury, ALF: Acute liver failure, CAD: Coronary artery disease, CLD: Chronic liver disease, CSF-PCR: Cerebrospinal fluid polymerase chain reaction, DL: Dyslipidemia, DM: Diabetes mellitus, H/O alcoholism: History of alcoholism, HT: Hypertension, LPD: Liver parenchymal disease, MAT: Microscopic agglutination test, NCCT: Non-contrast computed tomography, RT-PCR: Reverse transcription polymerase chain reaction

Cases	Age (years)	Sex	Community	H/O alcoholism	Occupation/Exposure	Comorbidities	Symptoms on admission	Leptospira IgM (NTU) Reference range: 9-11	MAT titer	CSF-PCR for leptospira	Malarial antigen test	Dengue antigen and antibody test	RT-PCR for COVID-19	Hepatitis A, B, C, D, E	Chest X-ray	Ultrasonography of abdomen	NCCT brain	CSF	Diagnosis	Duration of hospital stay (days)	Treatment received (intravenous)	Outcome
1	47	Male	Rural	Yes	Paddy field worker	DM, HT, DL	Fever, icterus, yellow urine, decreased appetite	16.2	Positive	-	Negative	Negative	Negative	Negative	Normal	CLD	-	-	Sepsis, LPD	<7	Ceftriaxone + Doxycycline	Recovery
2	36	Female	Rural	No	Cattle farming	None	Altered sensorium, fever, nausea/vomiting, abdominal pain	15.5	-	Positive	Negative	Negative	Negative	Negative	Normal	CLD	-	Pleocytosis	Meningitis, LPD	>7	Doxycycline	Recovery
3	48	Female	Urban	No	Field worker	DM, DL	Fever, body aches, nausea/vomiting	Kit positive	Positive	-	Negative	Negative	Negative	Negative	Normal	Hepatitis, Splenomegaly	-	-	Sepsis, AKI, Hepatitis	>7	Ceftriaxone + Doxycycline	Recovery
4	50	Male	Rural	Yes	Paddy field worker	DM, HT, CAD, CLD	Altered sensorium, icterus, breathing difficulty	30	Positive	-	Negative	Negative	Negative	Negative	Normal	Increased echogenicity in both kidneys	-	-	Sepsis, AKI, rash	>7	Ceftriaxone + Doxycycline	Death
5	41	Male	Urban	Yes	Sewage cleaner	HT, CLD	Abdominal distension, icterus, yellow urine, black stool	15.7	Positive	-	Negative	Negative	Negative	Negative	Normal	CLD, portal hypertension, splenomegaly	-	-	AKI, acute on CLD	<7	Doxycycline	Death
6	23	Male	Urban	No	Cattle farming	None	Fever, icterus, abdominal pain, abdominal distension	15.2	Positive	-	Negative	Negative	Negative	Negative	Normal	Hepatomegaly, edematous gallbladder	Normal	-	Sepsis, AKI, hepatitis, cholecystitis	>7	Meropenem + Doxycycline	Recovery
7	23	Male	Rural	No	Paddy field worker	HT	Altered sensorium, fever, productive cough, breathing difficulty	14.1	Positive	-	Negative	Negative	Negative	Negative	Patchy opacities in both lung fields	Hepatitis, splenomegaly	-	-	AKI, hepatitis, pneumonitis	>7	Meropenem + Doxycycline + Metronidazole + Steroid	Recovery
8	20	Male	Rural	Yes	Paddy field worker	None	Fever, icterus, decreased appetite	29.2	-	Positive	Negative	Negative	Negative	Negative	Normal	Normal	-	Pleocytosis	Sepsis, AKI, meningitis	<7	Meropenem + Doxycycline	Recovery
9	32	Male	Urban	Yes	Sewage cleaner	DM, DL, CLD	Altered sensorium, icterus, abdominal distension	19.7	Positive	-	Negative	Negative	Negative	Negative	Normal	CLD, Splenomegaly, Thickened Gall bladder	-	-	Acute on CLD, cholecystitis	<7	Meropenem + Doxycycline	Death
10	22	Male	Urban	No	Cattle farming	None	Fever, abdominal pain, nausea/vomiting, oliguria	11.9	Positive	-	Negative	Negative	Negative	Negative	Normal	Hepatomegaly, splenomegaly, gallbladder sludge	-	-	Sepsis, AKI, hepatitis, cholecystitis	<7	Meropenem + Doxycycline + Linezolid	Recovery
11	55	Female	Urban	No	Field worker	HT, DL, CAD	Fever, abdominal pain, decreased appetite, oliguria	17.2	Positive	-	Negative	Negative	Negative	Negative	Patchy opacity in the left lung field	Increased echogenicity in both kidneys	-	-	Sepsis, AKI, electrolyte imbalance, ALF, pneumonitis	>7	Meropenem + Doxycycline + Steroid	Recovery
12	28	Male	Rural	Yes	Paddy field worker	CLD	Fever, icterus, abdominal pain, vomiting, redness of eyes	26.1	Positive	-	Negative	Negative	Negative	Negative	Normal	Pancreatitis, Increased echogenicity in both kidneys	-	-	Sepsis, AKI, ALF, pancreatitis, conjunctival suffusion	>7	Meropenem + Doxycycline	Death

## Results

**Figure 1:**
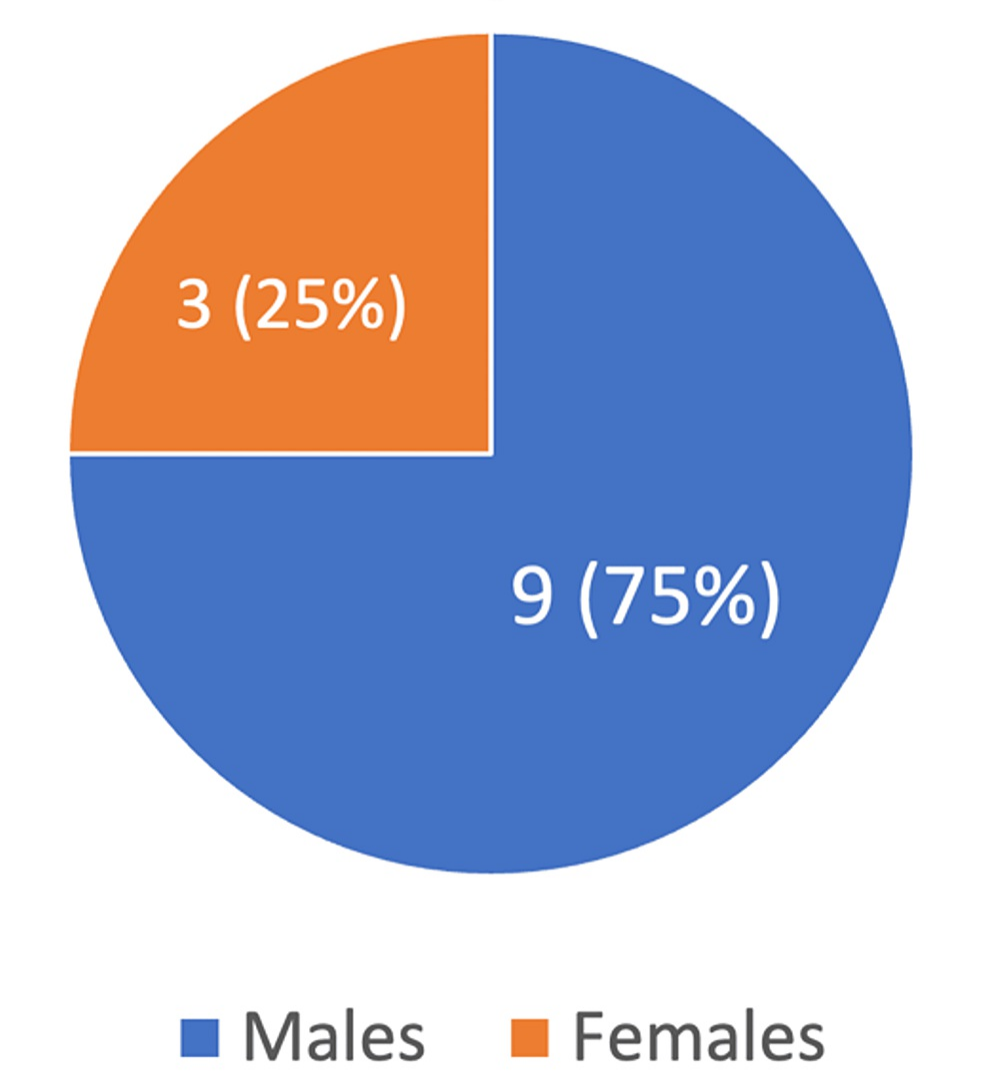
Pie chart showing gender distribution of cases.

A total of 12 cases were included in this case series after confirmation of leptospirosis. Out of 12 cases, there were nine (75%) males and three (25%) females (Figure 1).

The patients were divided into four groups according to their ages. It was found that the maximum number of cases were from the young age group of 20-29 years (41.66%), while the age group of 30-39 years and 50-59 years had the least number of cases (Table 2).

**Table 2:** Age distribution of cases.

Age group (years)	Number of cases	Percentage (%)
20-29	5	41.66
30-39	2	16.66
40-49	3	25.00
50-59	2	16.66

The most common symptom of presentation was fever with 11 (91.66%) cases, followed by icterus with 10 (83.33%) cases. Symptoms at presentation are described in Table 3.

**Table 3:** Symptoms of cases at presentation. The values in the table are represented in frequency (%).

Symptom	All cases (n=12)	Recovered (n=8)	Died (n=4)
Fever	11 (91.67%)	8 (100%)	3 (75%)
Icterus	10 (83.33%)	6 (75%)	4 (100%)
Abdominal pain	5 (41.67%)	4 (50%)	1 (25%)
Headache	5 (41.67%)	4 (50%)	1 (25%)
Body ache	5 (41.67%)	4 (50%)	1 (25%)
Nausea/vomiting	4 (33.33%)	3 (37.5%)	1 (25%)
Altered sensorium	4 (33.33%)	3 (37.5%)	1 (25%)
Oliguria	4 (33.33%)	2 (25%)	2 (50%)
Yellow urine	3 (25%)	2 (25%)	1 (25%)
Decreased appetite	3 (25%)	3 (37.5%)	0
Abdominal distension	3 (25%)	2 (25%)	1 (25%)
Breathing difficulty	2 (16.67%)	1 (12.5%)	1 (25%)
Black stool	1 (8.33%)	0	1 (25%)
Productive cough	1 (8.33%)	1 (12.5%)	0
Rash	1 (8.33%)	0	1 (25%)
Redness of eye	1 (8.33%)	0	1 (25%)

For the interpretation of data, we divided the patients into those who recovered and those who died and studied the association between risk factors and death using p-value and odds ratio. The demographic, clinical, laboratory, and treatment data along with their correlation with outcome using p-value and odds ratio are described in Table 4.

**Table 4:** Demographic, clinical, laboratory, and treatment data. ALP: Alkaline phosphatase, CKD: Chronic kidney disease, CLD: Chronic liver disease, COPD: Chronic obstructive pulmonary disease, CRP: C-reactive protein, CSF: Cerebrospinal fluid, H/O alcoholism: History of alcoholism, ICU: Intensive care unit, INR: International normalized ratio, PT: Prothrombin time, RBS: Random blood sugar, SGOT: Serum glutamic oxaloacetic transaminase, SGPT: Serum glutamic pyruvic transaminase. The values have been presented as median with interquartile range (IQR), and number with percentage (%) where appropriate. The relation is assessed by p-value and odds ratio for mortality with confidence intervals (CI). P-value <0.05 is considered statistically significant.

Variable	All cases (n=12)	Recovered (n=8)	Died (n=4)	P-value	Odds ratio for mortality (95% CI)
Median age (years)	34 (23-47.5)	29.5 (22.5-47.5)	36.5 (30-45.5)	0.68	1.66 (0.147-18.87) Odds of mortality for age >40 years
Sex					
Male	9 (75%)	5 (62.5%)	4 (100%)	0.28	5.72 (0.23-142.55) Odds of mortality male to female
Female	3 (25%)	3 (37.5%)	0		
Community					
Rural	6 (50%)	4 (50%)	2 (50%)	1	1.00 (0.09-11.02) Odds of mortality rural to urban
Urban	6 (50%)	4 (50%)	2 (50%)		
H/O alcoholism					
Yes	6 (50%)	2 (25%)	4 (100%)	0.05	23.40 (0.893-613)
No	6 (50%)	6 (75%)	0		
Comorbidities					
Hypertension	5 (41.66%)	3 (37.5%)	2 (50%)	0.68	1.66 (0.147-18.87)
Diabetes mellitus	4 (33.33%)	2 (25%)	2 (50%)	0.39	3.00 (0.23-37.67)
Dyslipidemia	4 (33.33%)	3 (37.5%)	1 (25%)	0.66	0.55 (0.03-8.08)
Coronary artery disease	2 (16.66%)	1 (12.5%)	1 (25%)	0.59	2.33 (0.106-50.98)
CKD	0	0	0		
COPD	0	0	0		
CLD	5 (41.66%)	1 (12.5%)	4 (100%)	0.04	24.00 (1.14-50.52)
Median duration of hospital stay (days)	10.5 (6-13)	8.5 (6-10.5)	11 (6.5-15.5)	0.68	0.60 (0.05-6.79)
ICU requirement	4 (33.33%)	1 (12.5%)	3 (75%)	0.05	21.00 (0.96-458.86)
Hemodialysis requirement	3 (25%)	0	3 (75%)	0.03	39.66 (1.27-1229.94)
Mortality	4 (33.33%)	-	-	-	-
Baseline laboratory values, median (IQR)					
Hemoglobin (g/dl)	9 (8.4-9.85)	9.1 (8.4-10.0)	8.9 (7-9.6)	0.58	-
Total leucocyte count (cells/cumm)	14350 (9350-19100)	15750 (11100-19350)	11800 (5750-16900)	0.39	0.33 (0.02-4.18) Odds of mortality for leukocytosis
Platelet count (cells/cumm)	135000 (78000-153000)	91500 (61500-169000)	148000 (120000-153000)	0.98	1.00 (0.06-15.98) Odds of mortality for thrombocytopenia
Total bilirubin (mg/dl)	9.35 (6.5-15)	7.05 (5.93-10.1)	16.9 (11.95-19.6)	0.01	1.80 (0.05-54.33)
Direct bilirubin (mg/dl)	7 (4.25-9.17)	4.8 (3.9-7.2)	9.17 (8.62-10.69)	0.01	1.80 (0.05-54.33)
SGOT (U/L)	82.5 (47-139.5)	60.5 (43-149.5)	107 (83-139.5)	0.82	3.46 (0.13-90.68)
SGPT (U/L)	47 (38.5-67.5)	56 (40-123.5)	42 (28-50)	0.11	0.60 (0.05-6.79)
ALP (U/L)	235.5 (128.5-532)	361 (128.5-725)	215 (145.5-235.5)	0.27	0.55 (0.03-8.08)
Albumin (mg/dl)	2.5 (2.1-3.2)	2.7 (2.3-3.2)	2.2 (1.95-3.2)	0.44	0.179 (0.005-5.678)
PT (seconds)	18 (13.6-26.5)	22 (15-27.5)	16.1 (13.1-22.5)	0.47	0.75 (0.03-17.50)
INR	1.7 (1-2.1)	1.7 (1.3-2.8)	1.2 (0.95-1.6)	0.35	0.25 (0.013-4.729)
Urea (mg/dl)	95 (64-179)	95 (64-179)	119 (41.1-274)	0.04	1.2 (0.073-19.63)
Creatinine (mg/dl)	3.3 (1-5.4)	3.1 (1-4.5)	4.6 (1.96-6.8)	0.04	1.2 (0.073-19.63)
Sodium (mEq/l)	135 (133-140)	136 (133-141)	133 (127.5-135.5)	0.37	4.00 (0.26-60.32)
Potassium (mEq/l)	3.4 (3.1-4.08)	3.4 (3.1-4.08)	3.65 (3.34-4.05)	0.86	0.75 (0.063-8.83)
Calcium (mg/dl)	8.97 (8.2-9.2)	8.75 (7.42-9.2)	8.97 (8.55-9.125)	0.63	0.66 (0.039-11.28)
CRP (mg/dl)	14.85 (12.95-16.45)	13.5 (11.95-14.9)	16.85 (15.75-18.20)	0.018	5.00 (0.34-72.77)
D-dimer (ng/ml)	2086.5 (1369.5-2641.5)	1648.5 (1179.5-2258.5)	2856.5 (2436.5-3058)	0.013	3.00 (0.21-42.62)
RBS (mg/dl)	98 (76-111)	99 (76-111)	98 (80.5-113)	0.66	2.00 (0.09-44.35)
Specific laboratory abnormalities					
Hypomagnesemia	1 (8.33%)	1 (12.5%)	0	-	-
High amylase level	1 (8.33%)	1 (12.5%)	0	-	-
High lipase level	1 (8.33%)	1 (12.5%)	0	-	-
Abnormal chest X-ray	2 (16.66%)	2 (25%)	0	-	-
CSF abnormality	2 (16.66%)	2 (25%)	0	-	-
Treatment received (intravenous), frequency (%)					
Doxycycline	2 (16.66%)	1 (12.5%)	1 (25%)	0.59	2.33 (0.106-50.98)
Ceftriaxone + Doxycycline	3 (25%)	2 (25%)	1 (25%)	0.71	0.50 (0.01-19.56)
Meropenem + Doxycycline	7 (58.33%0	5 (62.5%)	2 (50%)	0.57	0.40 (0.01-10.01)
Steroid	2 (16.66%)	2 (25%)	0	-	-

The median age was 36.5 (30-45.5) years in the group of patients who died which was higher in comparison to the group of patients who recovered, where the median age was 29 (22.5-47.5) years. The mean age of the patients who died was 37.75±9.81 years which was higher in comparison to those who survived which was 34.25±14.09 years. Males were more commonly affected comprising nine (75%) cases. There was an equal number of cases in rural and urban communities. A history of alcoholism was present in six (50%) of patients. The most common comorbidity was hypertension in five (41.66%) patients followed by diabetes and dyslipidemia in four (33.33%) patients. The median duration of hospital stay was 11 (6.5-15.5) days in patients who died which was higher than 8.5 (6-10.5) days in recovered patients. Only one (25%) patient survived, out of all patients who required intensive care treatment while no patient survived who required hemodialysis. Laboratory investigations were performed on all the patients. Complete blood counts in most cases revealed leukocytosis. The elevated urea and creatinine levels were not associated with small kidney size. Hyperbilirubinemia was present in most cases with a more evident rise in direct (conjugated) bilirubin levels. However, liver enzymes were either normal or moderately increased. High amylase and lipase levels were seen in one (8.33%) patient. Chest x-ray showed bilateral pulmonary infiltrates in two (16.66%) patients. Combination antibiotic therapy was required in 10 (83.33%) patients and had better outcomes than patients who were treated with single antibiotics. Steroid therapy was given to two (16.66%) patients, both of them recovered. History of alcoholism, preexisting chronic liver disease, jaundice, renal dysfunction, requirement of dialysis, and intensive care treatment were significantly associated with risk of death.

## Discussion

Leptospirosis is a disease with a wide variety of presentations, ranging from asymptomatic disease to severe illness. The signs and symptoms are non-specific and are similar to any febrile illness, which may complicate multiorgan failure. Background knowledge and clinical suspicion are very important for the timely diagnosis of this disease. A total of 12 patients of leptospirosis diagnosed by leptospira IgM antibody, MAT, and PCR test with either single or multiorgan system involvement were included in this study. There was a male preponderance in the study with nine (75%) cases and the male-to-female ratio was 3:1. This was similar to a study done by Sandhu et al. who also found male preponderance in cases of leptospirosis [5]. This male preponderance may be attributed to outdoor occupational activities increasing chances of exposure. The occupations of these patients included paddy field workers, sewage cleaners, and cattle farming. This finding was similar to a study by Katz et al. who found farmers, miners, sewage workers, fish farmers, and abattoir workers as occupational groups at higher risk of contracting leptospirosis [6]. Out of 12 cases, four (33.33%) patients died, while eight (66.67%) patients recovered. The median age of patients who died was 36.5 years which was higher than patients who recovered (median age 29.5 years). The median age in the mortality group was also higher in a study by Klement-Frutos et al., which is in concordance with our study [7]. Out of 12 patients, nine (75%) patients had renal involvement, eight (66.67%) patients had hepato-biliary system involvement, and seven (58.33%) patients had both renal and hepato-biliary system involvement. Sepsis was present in eight (66.67%) patients. Respiratory and central nervous systems were involved in two (16.66%) patients each. Among the least common involvement were the pancreas and skin, which were involved in one (8.33%) patient each.

For the interpretation of data, we divided the patients into those who recovered and those who died and studied the association with risk factors of death using p-value and odds ratio. Although the odds ratio for age and male sex were >1 (1.66 and 5.72 respectively), this association was not significant (p-value 0.66 and 0.28 respectively). We observed that factors like a history of alcoholism, the presence of chronic liver disease (CLD), hyperbilirubinemia, acute renal failure, the requirement of dialysis, and the requirement of intensive care significantly increased the odds of death (odds ratio >1, p-value <0.05). Other variables like age, sex, hypertension, diabetes mellitus, coronary artery disease, and dyslipidemia had no significant association with increased risk of mortality.

The most common symptom of presentation was fever with 11 (91.66%) cases, followed by icterus with 10 (83.33%) cases. This was consistent with studies by Goyal et al. and Sellors et al. in which fever was the most common symptom with 85.3% and 93% cases respectively [8,9]. There were an equal number of cases from rural (50%) and urban (50%) communities with each group having six cases. The association between community and outcome was also not statistically significant (p-value 1.0). History of alcoholism was present in six (50%) cases and the association with death was statistically significant (odds ratio 23.40, p-value 0.05). Similar to our study, Sethia et al. also found chronic alcohol intake as a significant risk factor for mortality in leptospirosis [10]. Pre-existing chronic liver disease was present in five (41.66%) cases, and it was significantly associated with death (odds ratio 24, p-value 0.04). Similar results were observed in studies by Goyal et al. and Somasundran et al., which showed an increased risk of death in leptospirosis in patients with liver cirrhosis [8,11]. Other comorbidities like hypertension, diabetes mellitus, dyslipidemia, and coronary artery disease were not significantly associated with death. Elevated total and direct bilirubin levels were significantly associated with death (odds ratio 1.8, p-value 0.01). This was similar to the study by Chang et al. which also showed the significant value of total and direct bilirubin in predicting mortality in leptospirosis [12]. Elevated serum urea and creatinine levels were also significantly associated with death (odds ratio 1.2, p-value 0.04). Studies by Sethia et al. and Panaphut et al. also found raised serum creatinine as a significant predictor of mortality in leptospirosis [10,13]. However, this finding was in contrast to a study by Goyal et al. who did not find raised serum creatinine as a significant predictor of mortality in leptospirosis [8].

Other laboratory parameters including serum hepatic transaminases, serum alkaline phosphatase (ALP), serum albumin, prothrombin time (PT), international normalized ratio (INR), total leucocyte and platelet counts, serum electrolytes, serum amylase and lipase levels and RBS levels were not significantly associated with death. In our study, serum alkaline phosphatase levels were proportionately much higher as compared to hepatic transaminases. However, there was no significant association between their levels and mortality. Similarly, hypoalbuminemia and deranged PT/INR values had no significant effect on mortality outcome which was in concordance with a study by Goyal et al. [8]. Anemia, leukocytosis, and thrombocytopenia had no significant impact on mortality in our study which was similar to findings in a study done by Al Hariri et al. [14]. Anemia in leptospirosis may occur from bleeding or hemolysis. Leukocytosis occurs as a response to bacterial infection. Thrombocytopenia in leptospirosis may occur by immune-mediated destruction, peripheral platelet consumption due to bleeding, and decreased production in bone marrow as described in the study by Davenport et al. [15]. In our study, hyponatremia and hyperkalemia were not associated with an increased risk of death which was similar to the observations made by Sethia et al. and Al Hariri et al. in their studies [10,14]. Our study showed the presence of hypomagnesemia in one patient who had acute renal failure. As elucidated by Khositseth et al. hypomagnesemia in leptospirosis occurs due to tubular dysfunction leading to urinary magnesium loss [16]. Our study included one case of acute pancreatitis with raised serum amylase and lipase levels. O’Brien et al. also described pancreatitis as a manifestation of leptospirosis in their study [17]. In our study, hypoglycemia was noted in 25% of cases in the death group, similar to a study by Kannan et al. where 28% of death cases had hypoglycemia [18]. Hypoglycemia in leptospirosis may be attributed to liver injury [18]. The levels of CRP and D-dimer were also not significantly associated with death. In our study CRP and D-dimer levels were higher than normal in both mortality and recovered groups, however, these values had no predictive value for mortality. These results were coherent with the findings of studies by Sellors et al. and Wagenaar et al. respectively [9,19].

Chest x-ray infiltrates and CSF abnormalities were present in two (16.66%) cases each and all of them survived and recovered. Chest x-ray infiltrates were due to pneumonitis, which was also depicted as a manifestation of leptospirosis in the study by Al Hariri et al. and it was a significant factor in predicting disease severity and mortality in that study [14]. In our study two cases presented with abnormal CSF findings which included raised leukocyte count (predominantly lymphocytes), elevated protein, and normal glucose levels. There were diagnosed as a case of aseptic meningitis which is also depicted as a manifestation of leptospirosis in the study by Wang et al. [20]. Intensive care treatment was required in four (33.33%) cases, out of which three cases died. Dialysis was required for acute renal failure in three (25%) cases and all three died. Both intensive care requirement (odds ratio 2.1, p-value 0.05) and dialysis requirement (odds ratio 39.66, p-value 0.03) were significantly associated with death. These results were similar to two other studies of leptospirosis. The study by Al Hariri et al. found that patients admitted to the intensive care unit had higher mortality [14] while Chang et al. found a higher risk of poor outcomes in patients with acute renal failure requiring dialysis compared to patients not requiring dialysis [12]. Median hospital stay was 11 days in cases who died while in recovered cases it was 8.5 days. However, the association was not statistically significant between number of days of hospital stay and death (p-value 0.68). Similarly, the study by Al Hariri et al. did not find any impact of prolonged hospitalization on outcomes [14]. All cases were treated with intravenous antibiotics along with supportive therapy. Treatment was started with intravenous doxycycline in all cases and later intravenous ceftriaxone or meropenem was added in cases where the response was poor with a single antibiotic or the patient had sepsis. Doxycycline alone was used in two (16.67%) cases, a combination of ceftriaxone and doxycycline was used in three (25%) cases, and a combination of meropenem and doxycycline was used in seven (58.35%) cases. The percentage of cases who died was lower in patients who received combination antibiotic therapy. Sellors et al. also used a combination of antimicrobials to treat leptospirosis in their study, which included penicillin and doxycycline [9]. However, they did not report the difference in the outcome of patients treated with single and combination antibiotic therapy. A short course of intravenous steroids was used as adjunctive treatment in two (16.67%) cases with pulmonary infiltrates, and both of them recovered. Pedro DM also used intravenous hydrocortisone in a case of leptospirosis with pulmonary involvement, which helped in the regression of disease and prevented mortality, thus supporting our findings [21].

Various other individual case reports have also documented the involvement of different organs in leptospirosis such as the liver, kidney, brain, pancreas, and lungs. Hyperbilirubinemia a common manifestation in leptospirosis is caused mainly due to raised levels of conjugated bilirubin and disturbance in bile excretion with intrahepatic cholestasis [1]. Acute kidney injury is common and can be non-oliguric or oliguric [22]. Electrolyte abnormalities include hyponatremia and hypokalemia. Loss of magnesium in urine is uniquely associated with leptospiral nephropathy. Altered mental status may be a sign of leptospiral meningitis [20]. Sometimes leptospirosis may also present as acute pancreatitis [23]. Death may occur due to internal bleeding mostly pulmonary hemorrhage [24]. Leptospira can involve one or more organ systems at the same time and cause serious complications or even worsen pre-existing diseases like chronic liver disease. The diagnosis of leptospirosis is often missed because of its diverse and non-specific clinical manifestations, overlapping features with other diseases, and also due to unavailability of proper diagnostic modalities and serologic tests in most hospitals. This prevents timely diagnosis and treatment that could minimize mortality in most cases. Hence there is a need to keep leptospirosis as a differential in patients presenting with non-specific signs and symptoms like unexplained or sudden elevation of direct bilirubin, acute kidney injury, pancreatitis, pneumonia, altered mental status, and multiorgan dysfunction.

Limitations

The study is limited by the small number of cases and a lack of control subjects. Casual association and generalization of the findings are difficult to make for a broad population. All patients were not followed up to see the long-term effects and impact on quality of life. Further multicentric studies with a large number of cases and controls will be required for the generalization of the results for a broader population 

## Conclusions

Leptospirosis is a bacterial infection whose symptoms and signs overlap with those of other acute febrile illnesses. Fever is the most common presenting symptom although a few patients can be afebrile. History of alcoholism, preexisting chronic liver disease, jaundice, renal dysfunction, requirement of dialysis, and need for intensive care management significantly increased the risk of death. Leptospirosis can be a cause of decompensation of preexisting compensated chronic liver disease. Patients with the above risk factors and comorbidities should be treated early and aggressively. Combination antibiotic therapy reduces mortality in severe disease This study will help raise awareness among physicians about various common, uncommon, and rare clinical presentations of leptospirosis so that early diagnosis and timely treatment can be done which will reduce mortality and improve outcome.
